# Intensity-Corrected Dual-Echo Echo-Planar Imaging (DE-EPI) for Improved Pediatric Brain Diffusion Imaging

**DOI:** 10.1371/journal.pone.0129325

**Published:** 2015-06-12

**Authors:** Kristen W. Yeom, Matus Straka, Michael Iv, Michael E. Moseley, Patrick D. Barnes, Stefan Skare, Samantha J. Holdsworth

**Affiliations:** 1 Department of Radiology, Lucile Packard Children’s Hospital, Stanford University, Stanford, CA, United States of America; 2 Institut für Radiologie und Nuklearmedizin, Kantonsspital Winterthur, Winterthur, Switzerland; 3 Department of Radiology, Stanford University, Stanford, CA, United States of America; 4 Department of Radiology, Lucas Center for Imaging, Stanford University, Stanford, CA, United States of America; 5 Clinical Neuroscience, Karolinska Institute, Stockholm, Sweden; University Medical Center Utrecht, NETHERLANDS

## Abstract

Here we investigate the utility of a dual-echo Echo-Planar Imaging (DE-EPI) Diffusion Weighted Imaging (DWI) approach to improve lesion conspicuity in pediatric imaging. This method delivers two ‘echo images’ for one diffusion-preparation period. We also demonstrate how the echoes can be utilized to remove transmit/receive coil-induced and static magnetic field intensity modulations on both echo images, which often mimic pathology and thereby pose diagnostic challenges. DE-EPI DWI data were acquired in 18 pediatric patients with abnormal diffusion lesions, and 46 pediatric patient controls at 3T. Echo1 [TE = 45ms] and Echo2 [TE = 86ms] were corrected for signal intensity variation across the images by exploiting the images equivalent coil-sensitivity and susceptibility-induced modulations. Two neuroradiologists independently reviewed Echo1 and Echo2 and their intensity-corrected variants (cEcho1 and cEcho2) on a 7-point Likert scale, with grading on lesion conspicuity diagnostic confidence. The apparent diffusion coefficient (ADC) map from Echo1 was used to validate presence of true pathology. Echo2 was unanimously favored over Echo1 for its sensitivity for detecting acute brain injury, with a mean respective lesion conspicuity of 5.7/4.4 (p < 0.005) and diagnostic confidence of 5.1/4.3 (p = 0.025). cEcho2 was rated higher than cEcho1, with a mean respective lesion conspicuity of 5.5/4.3 (p < 0.005) and diagnostic confidence of 5.4/4.4 (p < 0.005). cEcho2 was favored over all echoes for its diagnostic reliability, particularly in regions close to the head coil. This work concludes that DE-EPI DWI is a useful alternative to conventional single-echo EPI DWI, whereby Echo2 and cEcho2 allows for improved lesion detection and overall higher diagnostic confidence.

## Introduction

Diffusion MRI plays a key role in evaluating pediatric brain, not only for detecting acute injury but also for probing tissue changes that occur in pathologic conditions as well as in normal brain development [1–14]. In diffusion-weighted imaging (DWI), the image intensity relates (inversely) to the rate of microscopic water diffusion within a tissue voxel but also remains sensitive to other parameters, such as T1 and T2, and proton density. While detrimental T1 relaxation effects can be reduced by using a long repetition time (TR) in DWI, T2 effects are not easily mitigated due to the longer echo times (TE) brought by the presence of diffusion gradients. For this reason, the apparent diffusion coefficient (ADC) map—which removes the T2-weighting effect [15–16]—is typically calculated and used in conjunction with DW images to distinguish between lesions with reduced diffusion and those with edema (the latter sometimes referred to as “T2 shine-through”).

In a recent study, a parallel-imaging (PI) enhanced dual-echo (DE-EPI) DWI sequence was used to improve the overall fidelity and lesion conspicuity of acute/sub-acute/chronic infarcts in adult stroke patients [17]. Here, a second DW echo (Echo2) was acquired in the same TR as the first DW echo (Echo1), without impact on overall scan time for a typical set of DW imaging parameters used clinically. In that study the DWI images from Echo2 significantly improved acute diffusion-lesion detection while Echo1 was useful for generating higher SNR ADC maps [17]. An additional benefit of the DE-EPI method is that the two echoes can be utilized to remove transmit/receive coil-induced and static magnetic field (B0) intensity modulations, which might be confused as areas of diffusion restriction, and therefore potentially useful in the absence of coil-sensitivity calibration scans.

Typically, during image acquisition, patients lie in the radiofrequency coil such that the posterior brain region contacts the bottom part of the coil. As a result, the distance from the coil elements to the posterior and anterior parts of the brain may differ—the extent to which strongly depends on the patient’s head size, imaging coil diameter, and the way the patient head is padded in the coil. Since the signal intensity drops with squared distance from the coil, signal emanating from brain structures closer to the coil are often markedly increased. Particularly with higher channel coils—such as 8 to 32 channel-coils (that is, those with overall smaller coil elements), these individual coil elements receive a signal from a much smaller part of the brain, and thus the signal fall-off in regions farther from the coil surface is stronger. This effect may be even more pronounced when imaging children with smaller head sizes, and can be particularly problematic when the head is not centered in the coil.

The problem of the inhomogeneous sensitivity field of the neuroimaging coil is that the signal in every voxel is multiplied by an unknown factor consisting of two independent components: the coil sensitivity multiplied by the proton density. When reviewing the DW image, the image hyperintensity caused by restricted diffusion or edema cannot be easily distinguished from hyperintensity due to higher coil sensitivity; or, more often, the image intensity variability (due to sensitivity field variation) is so high such that some smaller lesions can remain obscured in the high-signal-areas (that are completely white due to the window-level/window-width setting during image review). Additionally, another source of erroneous signal on DWI is due to susceptibility-related image intensity variations (or signal ‘pile up’), which also can be confused for pathology. While these pile-ups and coil-sensitivity variations can be removed by a simple b1000/b0 ratio (that is, by creating an exponential ADC (eADC) map), this operation removes the T2-contrast imprinted on the original DWI—which can reduce the sensitivity of the DW image to lesions.

A way to extract and correct for the image intensity inhomogeneity problem is to make two measurements to estimate or compute the sensitivity-field, and subsequently remove this field from the original DWI. This can be accomplished by using a multi-echo acquisition to compute an artificial ‘intensity corrected’ DW image that is free of transmit and receive coil-sensitivity-field effects, proton density, T1 relaxation, and susceptibility-related image intensity variations. Currently, no study has investigated such potential added benefit of this artificial intensity correction that can be achieved from DE-EPI DWI. The goals of our study were two-fold: first, to devise an intensity correction method that removes the inhomogeneous sensitivity field from the DWI data; and secondly to assess the overall clinical performance of PI-enhanced DE-EPI DWI for pediatric brain using four sets of diffusion-weighted echoes (Echo1, Echo2, and their intensity-corrected complements, cEcho1 and cEcho2).

## Materials and Methods

### Study Cohort

Sixty two consecutive pediatric patients (median age 2 years; range 2 days to 18 years) who presented for assessment of intracranial pathology and obtained parallel-imaging (PI)-enhanced DE-EPI DWI at our children’s hospital from December 2012 and July 2013 were included after IRB approval from Stanford University and written informed consent was obtained from each subject’s parent(s). All patients obtained MRI exams at 3T (MR750; GE Healthcare, Milwaukee, Wisconsin) using an 8-channel head coil. Patients with dental hardware and exams with motion-degraded DE-EPI DWI were excluded. As part of routine MRI brain protocol, all patients also obtained the product DWI sequence, T1 and T2 FLAIR, T2 FSE, T2 FSE with fat suppression, T1 SPGR, and 2D GRE, arterial spin labeled perfusion, and in some cases, contrast-enhanced T1WI as deemed clinically necessary.

### Imaging Sequence and Reconstruction

**Fig 1:**
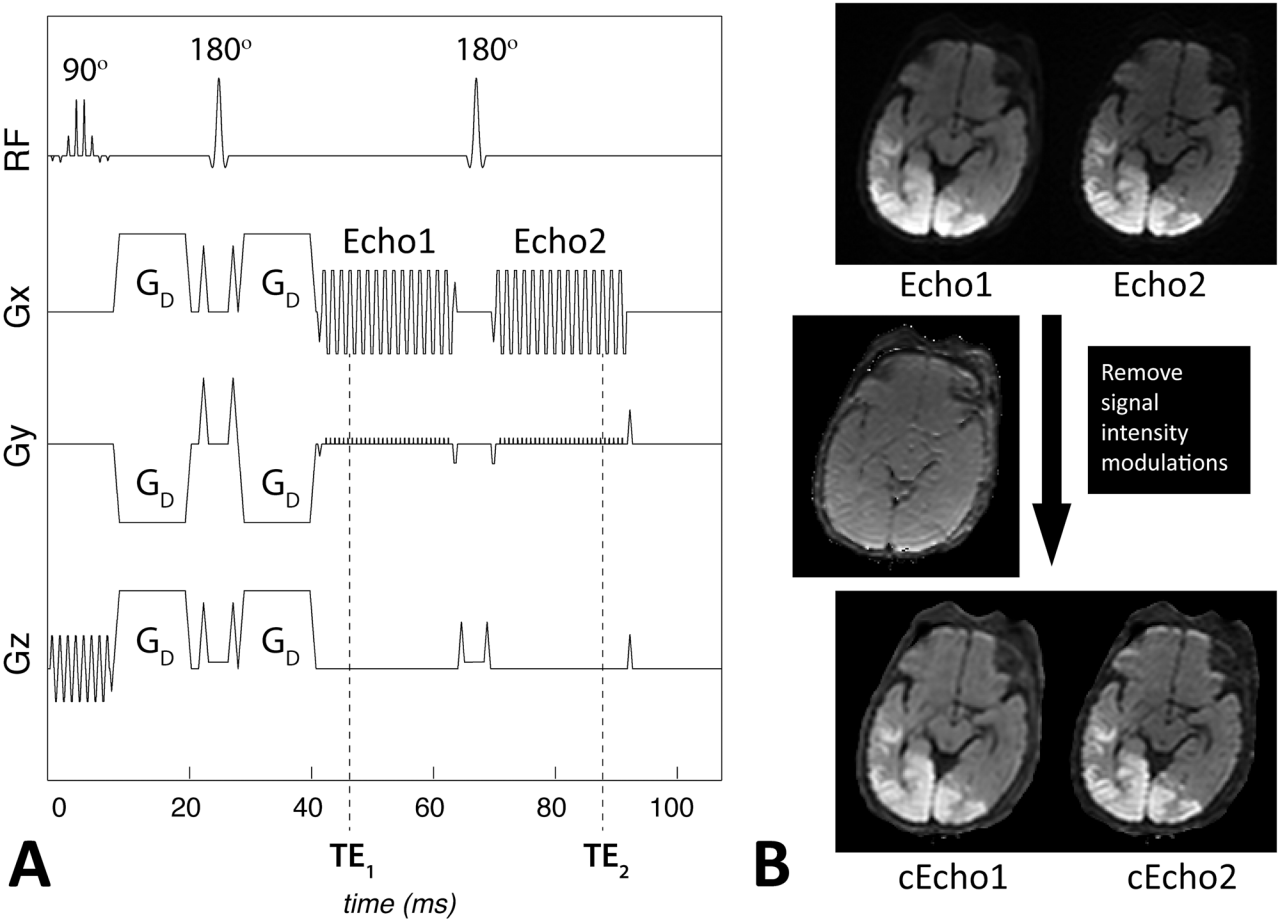
A. Our 1minute dual-echo Echo-Planar Imaging (DE-EPI) DWI pulse sequence corresponding to the acquisition of one slice, with timing and relative gradient and pulse amplitudes drawn approximately to scale. The diffusion (tetrahedral encoding) diffusion gradients, G_D_, are shown following slice selection with a 90° spectral spatial radiofrequency (RF) pulse. After the diffusion preparation period, Echo1 is acquired at TE_1_, followed by a 90° refocusing pulse and Echo2 at TE_2_. Note that the total acquisition time for one slice varies from 102–108ms depending on the FOV. B. Equivalent coil-sensitivity imprinted on Echo1 and Echo2 (here DWI) images allows one to extract and remove the contribution of receive coil and RF sensitivity, B0 susceptibility differences, T1, and proton density to the signal. Note that the resulting intensity-corrected images (cEcho1 and cEcho2) resemble that of an exponential ADC image with added T2 contrast. Data is acquired on a 9day old male infant with diffusion abnormality associated with hypoglycemia.

Fig 1A shows our in-house built PI-accelerated DE-EPI DWI sequence showing the acquisition of one slice, with timing and gradient amplitudes drawn approximately to scale. Here, two echoes are acquired—Echo1 represents the typical echo image that would be acquired clinically with such a PI-enhanced sequence, and Echo2 is the ‘less conventional’ Echo2 that is acquired succeeding a second 180° refocusing pulse. Patient data were acquired using the following imaging parameters: Generalized autocalibrating partially parallel acquisition (GRAPPA) [18–19] acceleration factor of R = 3, FOV = 20−24cm, acquisition matrix = 128 x 128, a 4mm/0mm slice thickness/gap, partial Fourier encoding with 24 overscans, TE_1_/TE_2_ = 46/85 ms, Stejskal Tanner diffusion preparation [20], tetrahedral encoding (4 diffusion directions) [21] with b = 1000 s/mm^2^, and 1 T_2_-w (b = 0) image. Each volume was acquired using 3 interleaves. The fully sampled b = 0 image was formed after combining the 3 interleaves and was used to enable the estimation of GRAPPA weights. These estimated GRAPPA weights were applied to all interleaves of all acquired volumes separately, including the b = 0 volume itself [17,22]. A TR of 4sec was used to keep T1 saturation effects small in the brain parenchyma (resulting in 92% and 99% T1 recovery at 3T, assuming a T1 = 1607ms/838ms for gray and white matter, respectively [23]), and a total scan time of 1 minute. Depending on selection of the FOV, the maximum number of slices permitted in a TR of 4s was between 36 (FOV = 20cm) and 39 (FOV = 24cm).

The first and second echo were corrected for signal intensity variation across the images using the following:

$${{Dc}_{i}\;=\;\frac{D_{i}}{C_{F}}\text{ for }i\;=\;1,2}^{\;}$$ 1

where *Dc*_*i*_ represents each corrected diffusion-weighted echo image (cEcho1 and cEcho2), *D*_*i*_ are the original diffusion-weighted echo images (Echo1 and Echo2), *C*_*F*_ is the Gaussian-filtered contribution of, *C*, which represents contributions to the signal intensity from T1 relaxation, proton density, static magnetic field (B0), coil and RF sensitivity as follows:

$$C\;=\;\frac{1}{\left(\frac{D_{1}}{{b0}_{1}}+\frac{D_{2}}{{b0}_{2}}\right)}\sum_{i\;=\;1}^{2}\frac{D_{i}}{e^{-{TE}_{i}R2}}$$ 2

where TE is the echo time, b0_i_ are the b = 0 s/mm^2^ images at the two echo times, and R2 is the relaxivity (1/T2) factor given by:

$$R2\;=\;\frac{1}{2\left({TE}_{2}-{TE}_{1}\right)}\left(\text{log}\left(\frac{{b0}_{1}}{{b0}_{2}}\right)+\text{log}\left(\frac{D_{1}}{D_{2}}\right)\right)$$ 3

where *D* are the DWI (b = 1000 s/mm^2^) images at the two echo times. A basic schematic representing the outcome of this correction method on a pediatric patient is shown in Fig 1B. Note that the intensity-corrected DWI maps are similar in appearance to eADC maps, however, they retain the T2-shine through component (this component is removed from the eADC maps since they are normalized by the b = 0 images).

The post-processing of the DE-EPI DWI data was performed using compiled and multi-threaded MATLAB code (version 7.8.0; Mathworks, Natick, MA, USA). The isotropic DWIs from the two echoes and their intensity-corrected variants (Echo1, Echo2, cEcho1, and cEcho2) were reconstructed and sent to our hospital image database (PACS). In addition, the isotropic ADC calculated from the b = 0 and b = 1000 s/mm^2^ of Echo1 was sent to PACS.

### Imaging Evaluation

#### Four diffusion echo sets

The four sets of diffusion-weighted echoes—Echo1, Echo2, cEcho1 and cEcho2—generated from DE-EPI DWI of the 62 patients were randomized and reviewed by two board-certified neuroradiologists with certificate of added qualification [Reviewer1 (KY) and Reviewer2 (MI), 8 and 3 years’ experience, respectively). The reviewers were blinded to the type of the diffusion echoes (Echo1, Echo2, cEcho1, cEcho2), as well as the clinical data, and final MRI-based diagnosis at all times; and they performed the review process independently and in multiple sessions.

The reviewers first assessed the images for presence of any lesion with reduced diffusivity that might reflect injury, ischemia, metabolic derangement, infection, hemorrhage, or cellular tumors. If present, the reviewers annotated the number and location of the lesions for each diffusion echo study. If the lesions were confluent and difficult to count due to large territory involvement, they were annotated as such and the number of brain locations (e.g. frontal, parietal, temporal, occipital lobes; brainstem; cerebellum) recorded.

The reviewers then graded the lesions for lesion conspicuity based on contrast between the lesion and the background tissue. If multiple lesions were present, a single score was generated reflecting combined impression of all the lesions. The reviewers also assessed for overall diagnostic confidence in making the decision process, taking into account the overall imaging quality, including contrast, SNR, and image distortion. For those exams that did not reveal lesions with reduced diffusivity, the reviewers only assessed for diagnostic confidence. For grading, the following seven-point Likert scale was used for grading: 1- nondiagnostic, 2- poor, 3- acceptable, 4- standard, 5- above average, 6- good, 7- outstanding.

Next, the reviewers performed a consensus-based analysis to determine if individual reviewer’s annotated lesions showed true reduced diffusivity rather than a T2-shine through effect based on corresponding low signal on the ADC map of Echo1 compared against the normal background brain tissue.

Reviewer1 also independently reviewed all 62 MRIs for presence of abnormal T2 or FLAIR intensity in the brain, blinded to the above DWI results.

#### Intensity correction

In the next step, Reviewers 1 and 2 performed a consensus-based review of cEcho1 and cEcho2 for all patients. The reviewers first evaluated the Echo1 and Echo2 dataset to identify potential artifacts attributable to intensity modulations; then examined cEcho1 and cEcho2 to determine adequate correction of erroneous intensity modulations (yes or no). In case of disagreement, a third board-certified neuroradiologist with certificate of added qualification (PB, >30 years experience) weighed in. The reviewers also assessed for any new changes or artifacts incurred by the intensity correction.

### Statistical Analysis

All statistical analyses were done with MATLAB code (version 7.8.0; Mathworks, Natick, MA, USA). Agreement amongst the two readers was assessed by a linearly weighted kappa statistic. Wilcoxon signed rank tests were used to assess the radiologist’s ratings.

## Results

### Clinical Findings

Of the 62 patients, 17 patients had pathologic lesions with reduced diffusivity confirmed by the ADC maps that also correlated with clinical presentation. Among these, 11 had intra-axial lesions, 4 had extra-axial lesions, and 2 had both intra-axial and extra-axial lesions. The patient demographics, clinical presentation, and the specific pathology of the 17 patients who presented with these lesions are summarized in Table 1.

**Table 1:** Patient demographics, clinical presentation, and specific neuropathology with reduced diffusion.

No.	Age	Sex	Clinical Presentation	Lesion Pathology
1	7 day	M	Hypoxic ischemic encephalopathy	Global brain injury
2	9 mo	F	Ventriculomegaly	Scalp hematoma
3	8 y	F	Chronic recurrent multifocal osteomyelitis	Skull/Facial bone osteomyelitis
4	10 y	F	Status epilepticus	Seizure edema
5	13 day	M	Liver disease, seizures	Seizure edema
6	17 mo	M	Glabellar lesion	Dermoid
7	63 wks	F	Liver disease, multi-organ failure	Ischemia
8	13 y	F	Sinonasal congestion	Skull base rhabdomyosarcoma
9	56 wks	M	Non accidental trauma	Global brain injury
10	8 wks	M	Non accidental trauma	Global brain injury
11	9 wks	F	Cardiac arrest	Cerebral infarct
12	9D	M	Hypoglycemia	Parietal, occipital, temporal brain injury
13	3 y	M	Trauma	Cerebral contusions
14	11 mo	M	Congenital heart disease	Cerebral infarct
15	16 mo	F	Biotin-thiamine responsive neurometabolic disorder	Diffuse brain injury/energy failure
16	11 y	F	Headache	Medulloblastoma
17	6 d	M	Decelerations at birth, possible maternal abruption	Parietal cortical infarct

Among the 17 patients, a total of 70 lesion sites were annotated by consensus review using all available echoes and ADC derived from Echo1. Specifically, 12 patients presented with 24 focal or discrete lesions. The remaining 5 patients presented with diffuse brain abnormality that involved a total of 42 specific anatomic brain regions, including various lobar structures, specific deep gray nuclei, brainstem, and cerebellar hemispheres, and additional 4 focal lesions.

The remaining 45 patients had either a normal MRI exam or other pathologic conditions that were not associated with reduced diffusivity. Examples of such conditions include: pineal cyst, old infarctions from ACTA2 mutation-related cerebral vasculopathy, posterior reversible encephalopathy syndrome, old brain injury, ventriculomegaly, arachnoid cyst, heterotopia, prior hemorrhage, Chiari I malformation, cooling post hypoxic-ischemic encephalopathy, tuberous sclerosis, headaches, epilepsy, congenital heart disease, trauma, and others.

### Intensity Correction

**Fig 2:**
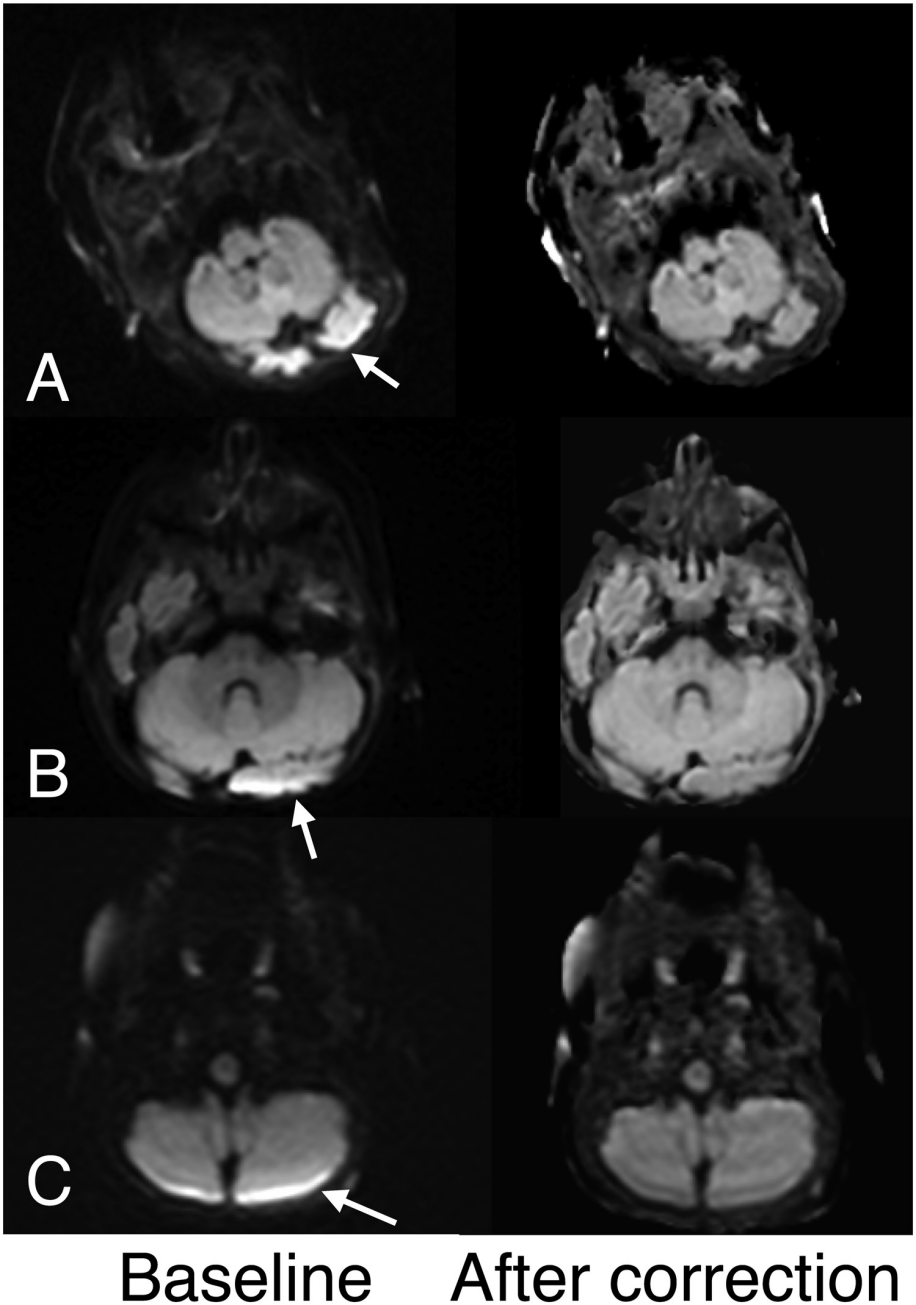
Examples of diffusion echoes before (baseline) and after intensity correction. A. Note that intensity variation (arrow) before correction is markedly improved after correction in this 7-day old female infant. B, C. Similar improvements are shown along the posterior fossae after intensity correction in a 4-month female infant (A) and a 17-month old male infant (B).

**Fig 3:**
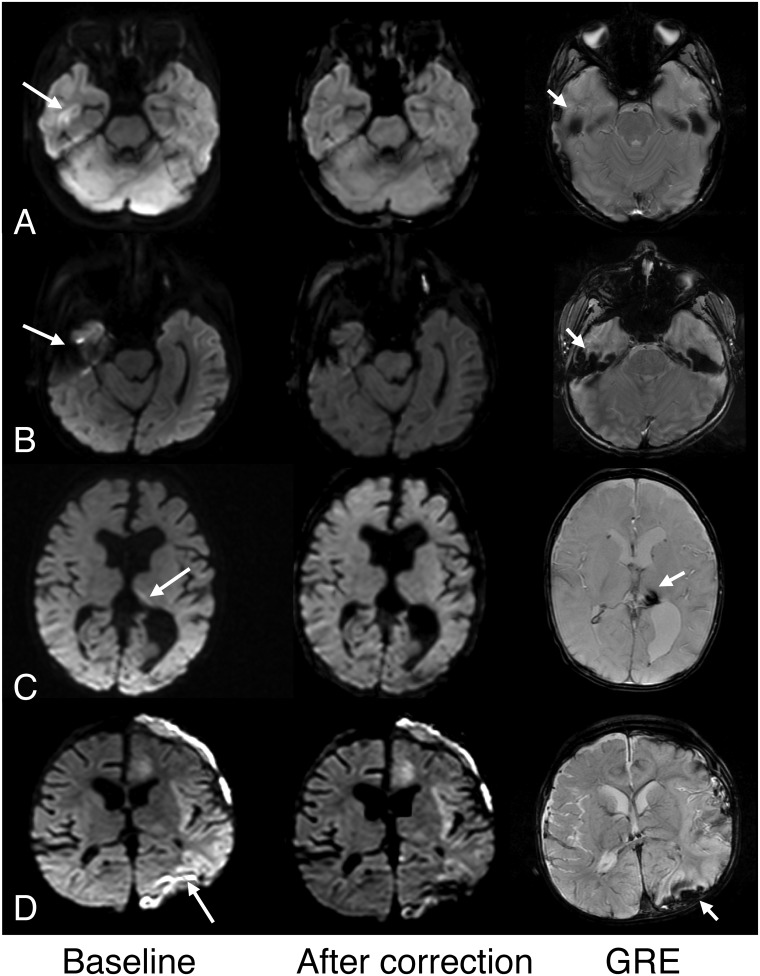
Examples of improved high intensity artifact associated with susceptibility after intensity correction. The columns represent baseline (Echo1 or Echo2); after correction (cEcho1 or cEcho2); and corresponding GRE images. A. High signal in the right temporal lobe cortex suggested possible pathology on Echo1 (shown here, arrow) and Echo2 (not shown) in this 6-year old girl but was considered an artifact from the temporal bone. Note improved more homogeneous brain signal on its intensity-corrected complement, cEcho1 (shown here) in both the temporal lobe and posterior brain regions. B. Similar improved susceptibility artifact is shown on cEcho1 compared to Echo1 (arrow) in a 6-year old boy. C. Susceptibility resulting from old hemosiderin is also mitigated on cEcho2 compared to Echo2 (arrow) in this 5-month old male infant with complicated birth history and remote intraventricular hemorrhage. D. Susceptibility artifact on Echo2 (arrow) related to subdural hemorrhage shown on GRE (short arrow) is improved after intensity correction on cEcho2 (shown here) in this 11 month old male infant with congenital heart disease and left cerebral infarction.

Intensity variations across the brain considered erroneous was present in all 62 pediatric patients on both Echo1 and Echo2; of these, the artifact was dominant in 53 patients such that it was considered to limit the diagnostic quality. In these patients, presence of such artifact posed additional challenges in assessing for presence of small subdural hemorrhage or ischemia of the occipital lobe or the cerebellum. In 3 children, no definite intensity differences were seen between the anterior or posterior brain regions but assessment was limited by reduced diffusivity occurring in the posterior brain regions (ischemia, hypoglycemia). Based on consensus review, cEcho1 and cEcho2 images corrected the artifacts found on Echo1 and Echo2, respectively, for all 53 patients. Examples are shown in Fig 2. The reviewers also identified reduced artifacts associated with susceptibility, most notable adjacent to temporal bones (Fig 3).

### Score Outcomes of the Diffusion-Weighted Echoes

Table 2 shows the mean and median ratings for reader assessment of lesion conspicuity and diagnostic confidence for Echoes 1 and 2, and their corrected variants, in all 62 subjects. The readers were in substantial agreement for their specific ratings (kappa = 0.74, 94%CI: 0.54–0.94).

**Table 2:** Mean and median (in brackets) ratings for reader assessment for Echoes 1 and 2, and their corrected variants.

	Patients with lesions (n = 17)		Patients without lesions (n = 45)	All subjects (n = 62)
	Lesion conspicuity	Diagnostic confidence	Diagnostic confidence	Diagnostic confidence
**Echo1**	4.4 (5)	4.3 (4)	4.4 (4)	4.4 (4)
**Echo2**	5.7 (6)	5.1 (6)	5.5 (6)	5.4 (6)
**cEcho1**	4.3 (4)	4.4 (4.5)	4.5 (4.5)	4.5 (4.5)
**cEcho2**	5.5 (6)	5.4 (6)	5.3 (6)	5.3 (6)

The interquartile range for every category in this table was equal to 1.

**Fig 4:**
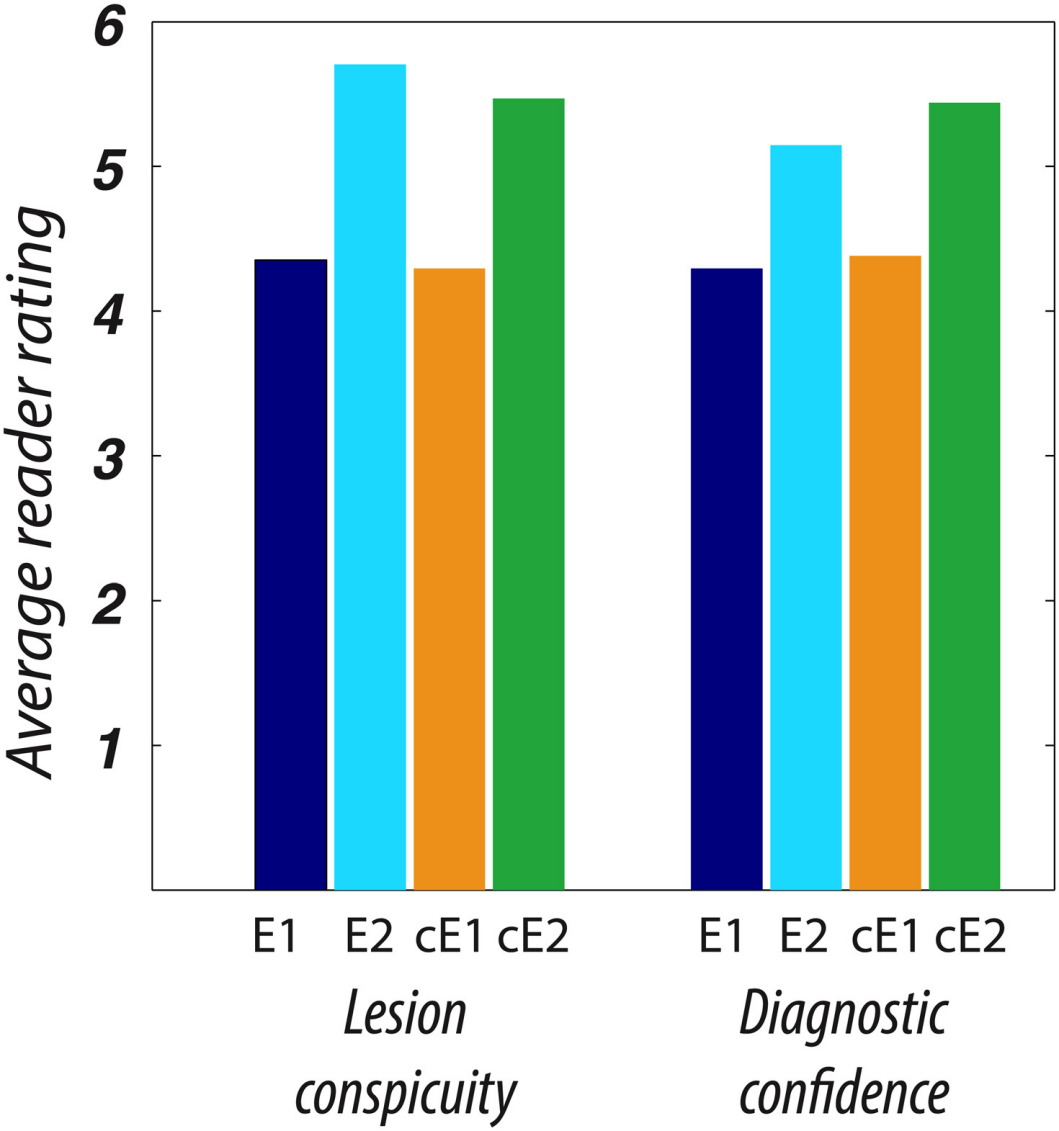
Stacked bar graph showing the grade value and frequency of Readers’ A and B assessment of lesion conspicuity and diagnostic confidence for Echo1 and Echo2 and their intensity corrected variants (cEcho1 and cEcho2) for patients that had reduced diffusivity.

**Fig 5:**
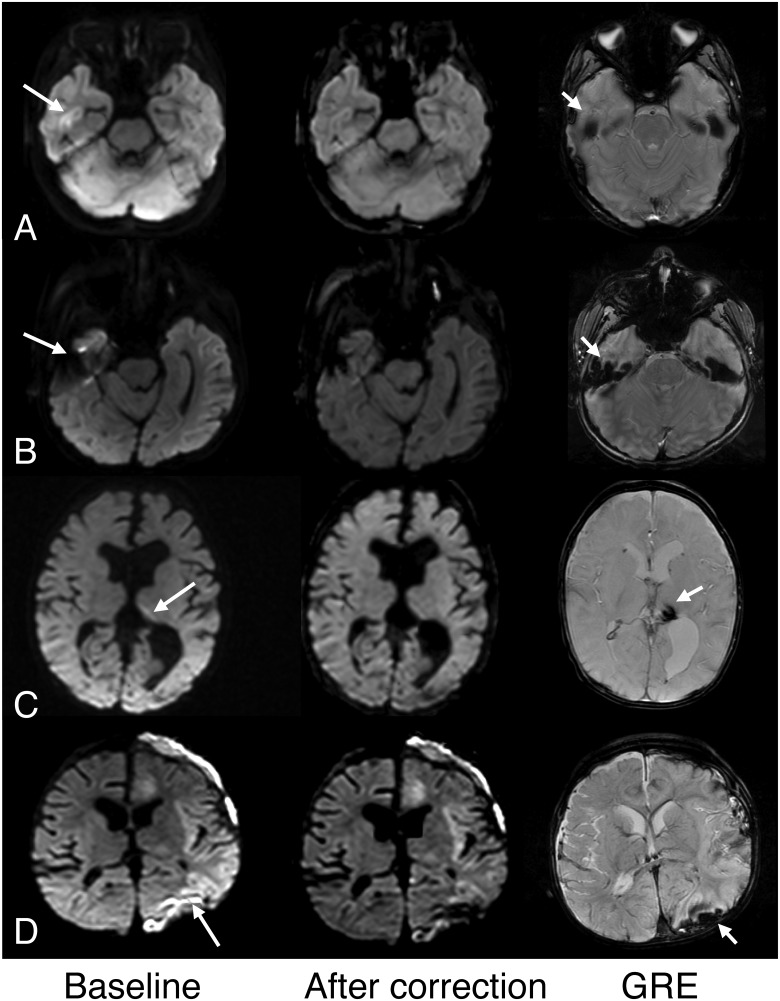
Examples of superior lesion conspicuity on Echo2 or its intensity corrected complement, cEcho2 compared to Echo1 or cEcho1. A. Note improved conspicuity of cerebral contusions (arrows) in this 3-year old boy who fell from a 2-story building on Echo2 compared to Echo1. B. Reduced diffusivity from hypoxic-ischemic encephalopathy is more distinctly depicted on Echo2 (long arrow) compared to Echo1 in a 7-day old male infant who was cooled for neonatal encephalopathy. Note also focal reduced diffusivity of right cerebral peduncle (short arrow) is also better delineated on Echo2. C. Reduced diffusivity from hypoglycemia is more conspicuous on the cEcho2 image (arrows) compared to cEcho1 in a 9-day old male infant. D. Similar findings are also noted for this 8-week old male infant presenting with brain injury from non accidental trauma (arrows).

Fig 4 shows the frequency of ratings for reader assessment of lesion conspicuity calculated on the 17 subjects with pathologic lesions with reduced diffusivity, and of diagnostic confidence on all subjects. Echo2 images were rated significantly higher than Echo1, with a mean respective lesion conspicuity of 5.7/4.4 (p < 0.005) and diagnostic confidence in patients with lesions of 5.1/4.3 (p = 0.025). cEcho2 images were rated significantly higher than cEcho1, with a mean respective lesion conspicuity of 5.5/4.3 (p < 0.005) and diagnostic confidence of 5.4/4.4 (p < 0.005). Examples illustrating the higher lesion conspicuity for Echo2 and cEcho2 images are shown in Fig 5.

For the same 17 cases, the mean overall scores for Echo2 were rated only slightly higher than for cEcho2 on lesion conspicuity, while the converse was true for diagnostic confidence. Among the negative cases, diagnostic confidence scored slightly higher for Echo2 versus cEcho2. However these findings were not statistically significant. Similarly, there was no statistical difference between the mean grading of Echo1 and cEcho1.

### False positive and Negative Cases

**Fig 6:**
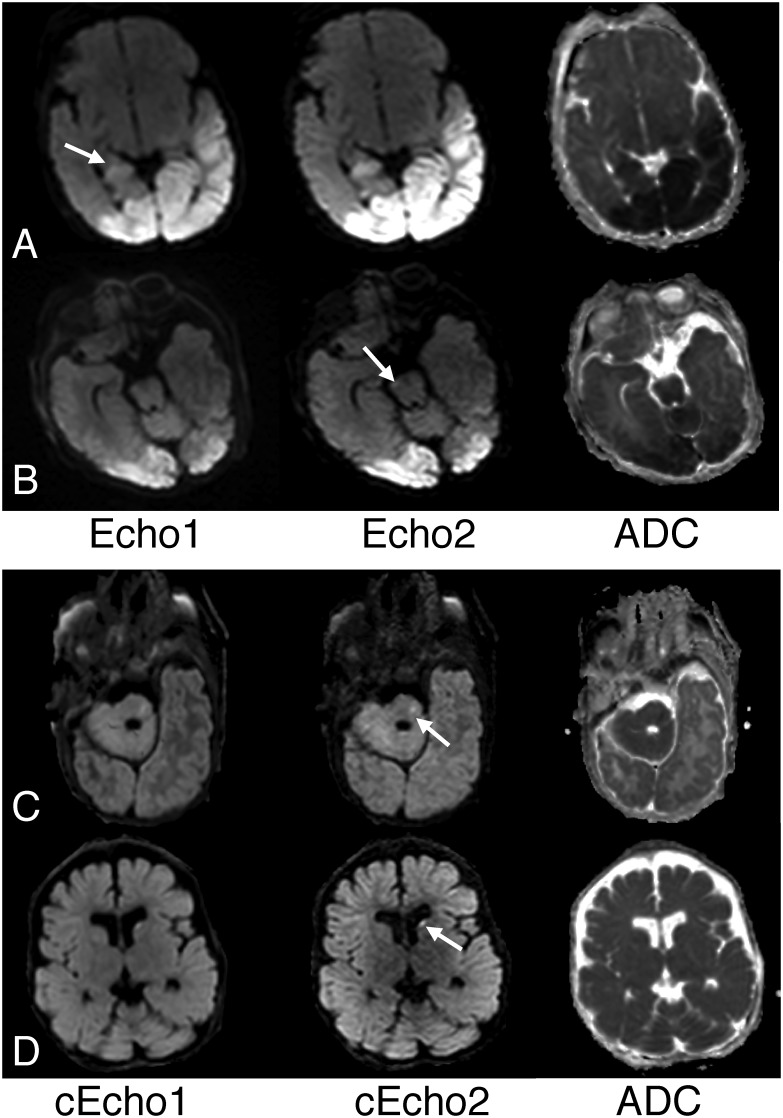
Examples of false negatives, or lesions missed by either of the reviewers. A. While both reviewers identified various lobar brain regions of reduced diffusivity affected by hypoglycemia in this 9-day old male infant, right hippocampal involvement was noted on Echo2 and cEcho2 (not shown) but not for Echo1 or cEcho1. In retrospect, subtle high signal in right hippocampus on Echo1 (arrow) is identified but less conspicuous compared to Echo2. B. Same patient as Fig 5B where focal lesion in right cerebral peduncle was overlooked by both reviewers, perhaps due to small size, but identified by Reviewer1 on both Echo2 (arrow) and cEcho2 (not shown). C. Focal lesion in the left pons was identified by both reviewers on cEcho2 (arrow) and Echo2 (not shown) and but missed on Echo1 and cEcho1 in this 9-week old infant with cardiac arrest. D. Focal embolic infarct in the left caudate of a 63-week old female infant was missed on Echo1 by both reviewers but detected on cEcho2 (arrow), as well as Echo2 (not shown) and cEcho1.

Of the 70 lesion sites, initial blinded review missed, or resulted in a total false negative of, 12 lesions (Reviewer1 = 8 lesions; Reviewer2 = 11 lesions). For both reviewers, highest number of false negatives occurred for Echo1 and cEcho1. Examples are shown in Fig 6.

**Fig 7:**
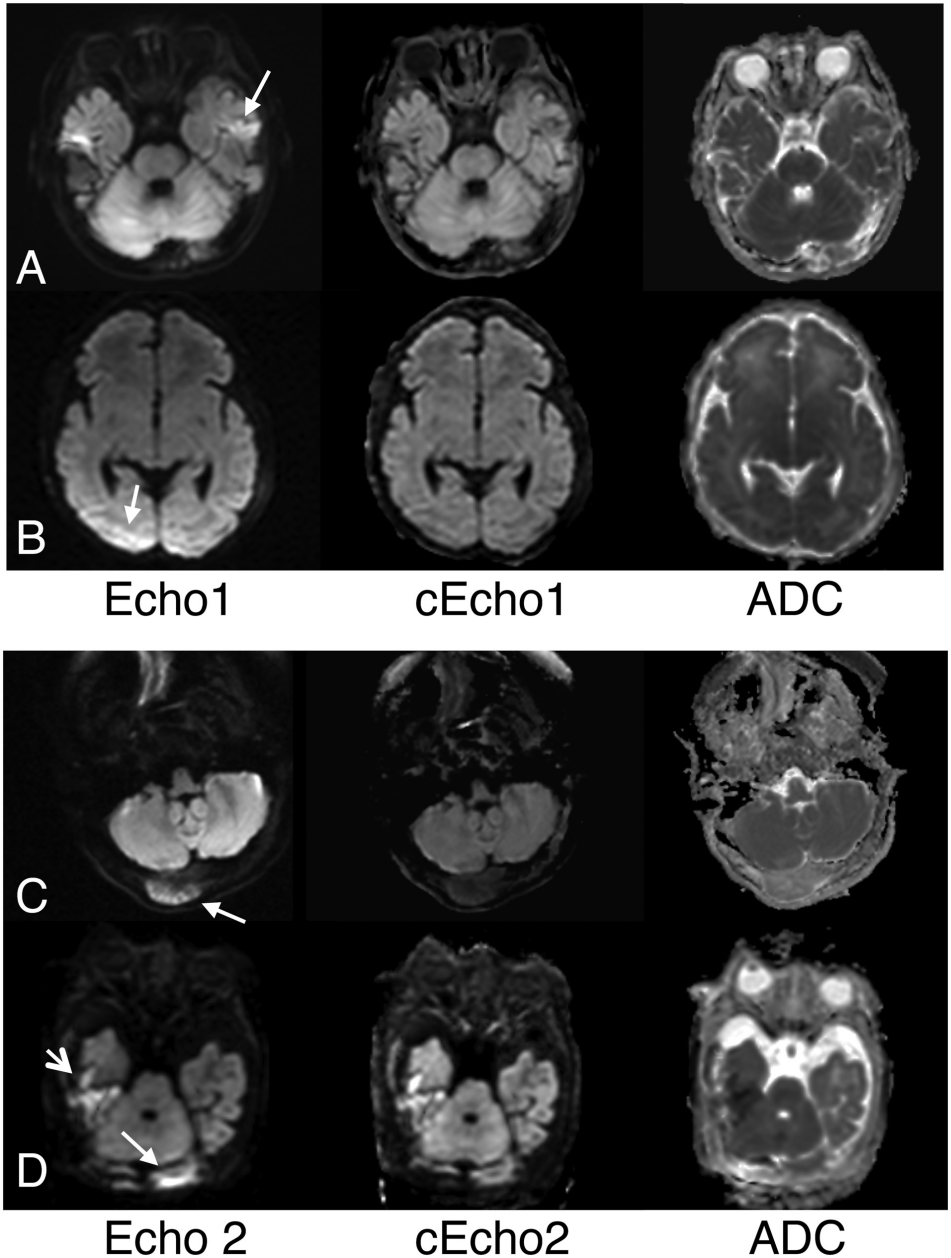
Examples of false positives lesions. The majority of these cases resulted from coil-induced brain signal variations in the posterior regions and/or susceptibility-related high signal present in baseline echoes (Echo1 and Echo2). A. Left temporal lobe cortical high signal (arrow) seen on multiple axial images on Echo1 raised possibility of pathology given other scattered lesions in the brain in this 16-month female with a biotin-thiamine responsive basal ganglia disease, a neurometabolic disorder. This was not associated with low signal on ADC and was considered to reflect combination of temporal bone artifact and T2-shine through from an underlying T2/FLAIR high intensity present in this child. Note high signal intensity associated with bilateral temporal bone susceptibility is markedly mitigated on its intensity-corrected complement, cEcho1. B. Asymmetric high signal was seen at baseline on Echo1 (arrow) and Echo2 (not shown) images, raising suspicion of pathology in this 7-day old infant cooled for neonatal encephalopathy. However, this represented erroneous intensity modulations and was without associated low ADC signal. cEcho1 showed improved signal variation in the posterior brain region. C. The reviewers initially thought the right occipital bone tumor (sarcoma metastasis) in this 16-year old girl showed reduced diffusivity on both Echo2 (arrow) and Echo1 (not shown), but this was found to represent a T2-shine through effect from tumor high water content. Note that cEcho2 better reflects the non-cellular nature of the tumor. D. High signal along the left posterior brain on Echo2 (arrow) and Echo1 (not shown) was suspected to represent either hemorrhage or acute parenchymal lesion in this 13-day old male infant with coagulopathy and liver disease and hemorrhagic collection elsewhere in the brain (short arrow). However, the left posterior brain high signal arose from inhomogeneous signal modulations and not true pathology, and was found to be corrected on both cEcho1 (not shown) and cEcho2 images.

Of all 62 patients, the reviewers identified a total of 9 lesions (Reviewer1 = 5 lesions; Reviewer2 = 5 lesions) that were not validated by ADC and therefore deemed false positives. They occurred in two subjects with no abnormal MRI findings and seven patients who had reduced diffusivity elsewhere in the brain. All false positives occurred on Echoes 1 and 2 reads and none for cEchoes 1 and 2. Examples are shown in Fig 7. A summary of false negative/positive cases based on individual echo dataset is shown in Table 3.

**Table 3:** A summary of false negative and positive cases stratified by the individual echo dataset.

	Reviewer 1		Reviewer 2	
	False Negative (n = 8)	False Positive (n = 5)	False Negative (n = 11)	False Positive (n = 5)
**Echo1**	8	5	11	5
**Echo2**	1	3	3	3
**cEcho1**	7	0	9	0
**cEcho2**	0	0	3	0

### T2WI/FLAIR Abnormality

Of the 62 patients, 12 patients showed pathologic T2WI/FLAIR high signal abnormality in the brain reflecting edema or prior injury and not associated reduced diffusivity. An additional 13 patients showed T2WI/FLAIR high signal in the white matter due to age-related immaturity and undermyelination. Among these, one case showed high signal on Echo1 (but not other diffusion datasets) corresponding to FLAIR high intensity and without low signal on ADC (Fig 6A), that suggested a T2 shine-through effect.

## Discussion

Here we showed that our method for erroneous signal intensity correction using a PI-enhanced DE-EPI DWI technique is feasible, and that Echo2 and cEcho2 derived from DE-EPI DWI may be alternatives to conventional single-echo echo planar DWI for improved lesion detection and overall diagnostic confidence. While a prior study has shown heighted sensitivity of DE-EPI DWI technique to acute/subacute infarcts in adults [17], no study has leveraged on the inherent advantage of DE-EPI DWI to permit intensity-correction. Furthermore, this is the first study to investigate the potential benefit of DE-EPI DWI in children in whom T1 and T2 changes that rapidly occur in the early developmental years [24–25]. Also, while children may present with strokes, in pediatric neuroimaging, DWI is often used to probe more diverse forms of tissue injury and energy failure that might manifest with a wider range of reduced diffusivity and in some cases, reversible tissue changes, as well as congenital or acquired pathologies unique to children.

We found that both Echo2 and its intensity-corrected complement, cEcho2, robustly identified various pathologies with reduced diffusivity and with significantly higher scores for lesion conspicuity and diagnostic confidence compared to either Echo1 or cEcho1. While Echo2 generated fewer false positive reads compared to Echo1, there were no false positives for cEcho2. We also found a higher rate of false negative reads for Echo1 or cEcho1—attributed to poor lesion conspicuity, particularly for small lesions or lesions arising in the brain periphery.

Our findings are consistent with a study that reported a higher Echo2 sensitivity to acute stroke in adults due to its additional T2 contrast compared with Echo1 [17], and in a study that incidentally observed improved conspicuity of ischemic lesions bought by high acquisition matrices (thus long TEs) [26]. This study confirms its potential benefit of DE-EPI DWI in children in the workup of other pathologies with reduced diffusivity, such as neonatal encephalopathy, nonaccidental/accidental brain trauma, seizure edema, metabolic disorders, cellular tumors, as well as extra-cranial lesions such as dermoid/epidermoid. While majority of our cohort comprised young children (33 of 62 children aged 2 and younger), white matter T2 or FLAIR high intensity related to higher water content from immaturity/undermyelination did not adversely impact diagnostic capability of Echo2 or cEcho2 with a longer TE. In fact, no case of a T2 shine-through occurred except for Echo1 of one patient who had high FLAIR signal associated with a metabolic disorder.

We also found that signal variations that might mimic pathology were common, occurring in 85% of our cohort, possibly due to small head size of our young patients (median age 2 years) that might result in a more discrepant anterior and posterior brain distance from the coil. In fact, presence of such artifact tended to reduce reviewer diagnostic confidence and was the main source of false positives for Echo1 or Echo2. To our knowledge, this is the first study to describe a method for intensity correction that utilizes the advantages of a DE-EPI technique. Intensity correction was effective when such artifacts were present; and notably, there were no cases of false positives for cEcho1 or cEcho2 in a blinded review.

Another advantage of the intensity correction method is the reduction of bright susceptibility-related artifacts at sites prone to off-resonance effects (e.g. skull base, hemorrhages, surgical sites, etc.) (Fig 3). This susceptibility-differences-induced pile-up of the signal in the image is a feature of the EPI method and present in the images regardless of the underlying diffusion or diffusion weighting—and may occasionally confound the diagnostic search. While the intensity correction method introduced here does not improve the underlying geometric fidelity of the EPI images, incorporating the intensity correction method may improve lesion detection in susceptibility-prone brain regions that cause confounding signal pile-up, providing a potential added clinical benefit.

Despite the lack of a clear winner, our study identified potential benefits of both Echo2 and cEcho2 that may be tailored for individual clinical need and at no additional scan time. Also, our results suggest that cEcho2, associated with fewest false negatives/positives, superior capacity for lesion detection, and decreased artifacts associated with coil-sensitivity and susceptibility, may be serve a useful future role for diffusion imaging. Using the DE-EPI DWI approach, we suggest the combined use of Echo2 and cEcho2 for improved lesion detection (and with fewer false positives); for ADC, we suggest the use of ADC map calculated from Echo1, to confirm suspected diffusion pathology due to its higher SNR compared to ADC generated from Echo2.

The reader may point out that the computed ADC maps are also free of the erroneous intensity modulation effect—since this effect is identical in both diffusion-weighted (DWI or b > 0) and diffusion-unweighted (b = 0) images—and thus could be used in place of the DWI. However, the interpretation of brain pathologies cannot rely on ADC maps only, for two confounding effects: (1) Due to possible ADC-pseudonormalization where the ADC returns to or remains at levels typical for normal tissue, pathology may be inconspicuous. This happens if the reduction in diffusion rate (which causes hyperintensity in the DW image) is counteracted by high water contents in the tissue (which causes hyperintensity in the b = 0 image); thus the pathologic tissue is not distinctly identifiable in the ADC maps as pathologic; (2) Due to the lower SNR of the ADC maps, many small or tiny pathologies in the brain are likely to remain obscured or unidentified. For these reasons, review of the original DWI b>0 has strong advantages and remains the primary method of diagnostic evaluation.

An alternative method for computing and correcting for the sensitivity-field is through the use of two different coils (an explicit approach). In this case, one coil (e.g. a body coil) must itself have a homogenous sensitivity field that can be used to map the sensitivity field of the second coil (here, the neuroimaging coil), albeit at low resolution. This body-coil estimated sensitivity field calibration can then be used to correct the higher-resolution DWI scan from the neuroimaging coil. However, this approach requires two scans (for each coil), thus prolonging the exam duration. This approach also requires that the patient remains motionless during both scans—as the sensitivity field can be only estimated in regions where actual MRI-signal-generating body tissue is present—and therefore is suboptimal for pediatric imaging. On the contrary, the intensity-corrected DE-EPI approach is implicit, and mostly guarantees the computation of a motion-free sensitivity field since the two echoes (from which this the field is calculated) are acquired within ~40 milliseconds of each other.

We recognize certain limitations. First, the sample size of subjects with reduced diffusivity was relatively small, attributed to rarer occurrence of strokes in children compared to adults. On the other hand, children presented with more diverse pathology manifesting with reduced diffusivity, which helped to assess potential benefit of DE-EPI DWI in assessing pathology other than stroke. Despite the small sample size, we identified 70 lesions/lesion sites, and investigated additional 45 subjects without diffusion abnormality, that were used to assess advantages or potential pitfalls of DE-EPI DWI.

## Conclusion

In this study, we present a method for reducing erroneous signal intensity modulations on DWI using a PI-enhanced DE-EPI DWI technique that could benefit DWI assessment of young children. Our results also show Echo2 and its intensity-corrected complement, cEcho2, derived from DE-EPI DWI may be alternatives to the use of the conventional single-echo EPI DWI approach for improved lesion detection and overall diagnostic confidence.
